# Structure-guided Alterations of the gp41-directed HIV-1 Broadly Neutralizing Antibody 2F5 Reveal New Properties Regarding its Neutralizing Function

**DOI:** 10.1371/journal.ppat.1002806

**Published:** 2012-07-19

**Authors:** Javier Guenaga, Richard T. Wyatt

**Affiliations:** 1 IAVI Neutralizing Antibody Center at The Scripps Research Institute, La Jolla, California, United States of America; 2 Department of Immunology and Microbial Science, The Scripps Research Institute, La Jolla, California, United States of America; University of Zurich, Switzerland

## Abstract

The broadly neutralizing HIV-1 antibody 2F5 recognizes an epitope in the gp41 membrane proximal external region (MPER). The MPER adopts a helical conformation as free peptide, as post-fusogenic forms of gp41, and when bound to the 4E10 monoclonal antibody (Mab). However, when bound to 2F5, the epitope is an extended-loop. The antibody-peptide structure reveals binding between the heavy and light chains with most the long, hydrophobic CDRH3 not contacting peptide. However, mutagenesis identifies this loop as critical for binding, neutralization and for putative hydrophobic membrane interactions. Here, we examined length requirements of the 2F5 CDRH3 and plasticity regarding binding and neutralization. We generated 2F5 variants possessing either longer or shorter CDRH3s and assessed function. The CDRH3 tolerated elongations and reductions up to four residues, displaying a range of binding affinities and retaining some neutralizing capacity. 2F5 antibody variants selective recognition of conformationally distinctive MPER probes suggests a new role for the CDRH3 loop in destabilizing the helical MPER. Binding and neutralization were enhanced by targeted tryptophan substitutions recapitulating fully the activities of the wild-type 2F5 antibody in a shorter CDRH3 variant. MPER alanine scanning revealed binding contacts of this variant downstream of the 2F5 core epitope, into the 4E10 epitope region. This variant displayed increased reactivity to cardiolipin-beta-2-glycoprotein. Tyrosine replacements maintained neutralization while eliminating cardiolipin-beta-2-glycoprotein interaction. The data suggest a new mechanism of action, important for vaccine design, in which the 2F5 CDRH3 contacts and destabilizes the MPER helix downstream of its core epitope to allow induction of the extended-loop conformation.

## Author Summary

Host antibodies raised in response to acute viral infection are often protective to second exposure. However, in the less frequent examples of chronic infection, in which the virus actively replicates for prolonged periods, host immunity can impact on viral characteristics by applying selective pressures upon progeny. Such a dynamic process is exemplified by the extremely variable and pathogenic human immunodeficiency virus type 1 (HIV-1). Relatively infrequently, antibodies are elicited during infection that can neutralize a diverse array of this malleable pathogen. Hence, studies which elucidate such antibodies are elevated in importance if the pathogen causes human suffering, yet no vaccine exists. Here, we describe a new property of the broadly neutralizing antibody, 2F5, which is directed to a conserved region of the HIV-1 surface protein near the lipid membrane. Through mutagenesis of the antibody and subsequent functional analysis, we present data that suggest a model in which the antibody first binds downstream of its known core epitope in a two-step process not directly involving the lipid membrane. Such studies may better elucidate the not yet defined details of virus-to-cell fusion by which viral DNA enters host target cells. Additionally, such analysis reveals 2F5 binding specificities, important for future vaccine designs.

## Introduction

**Figure 1:**
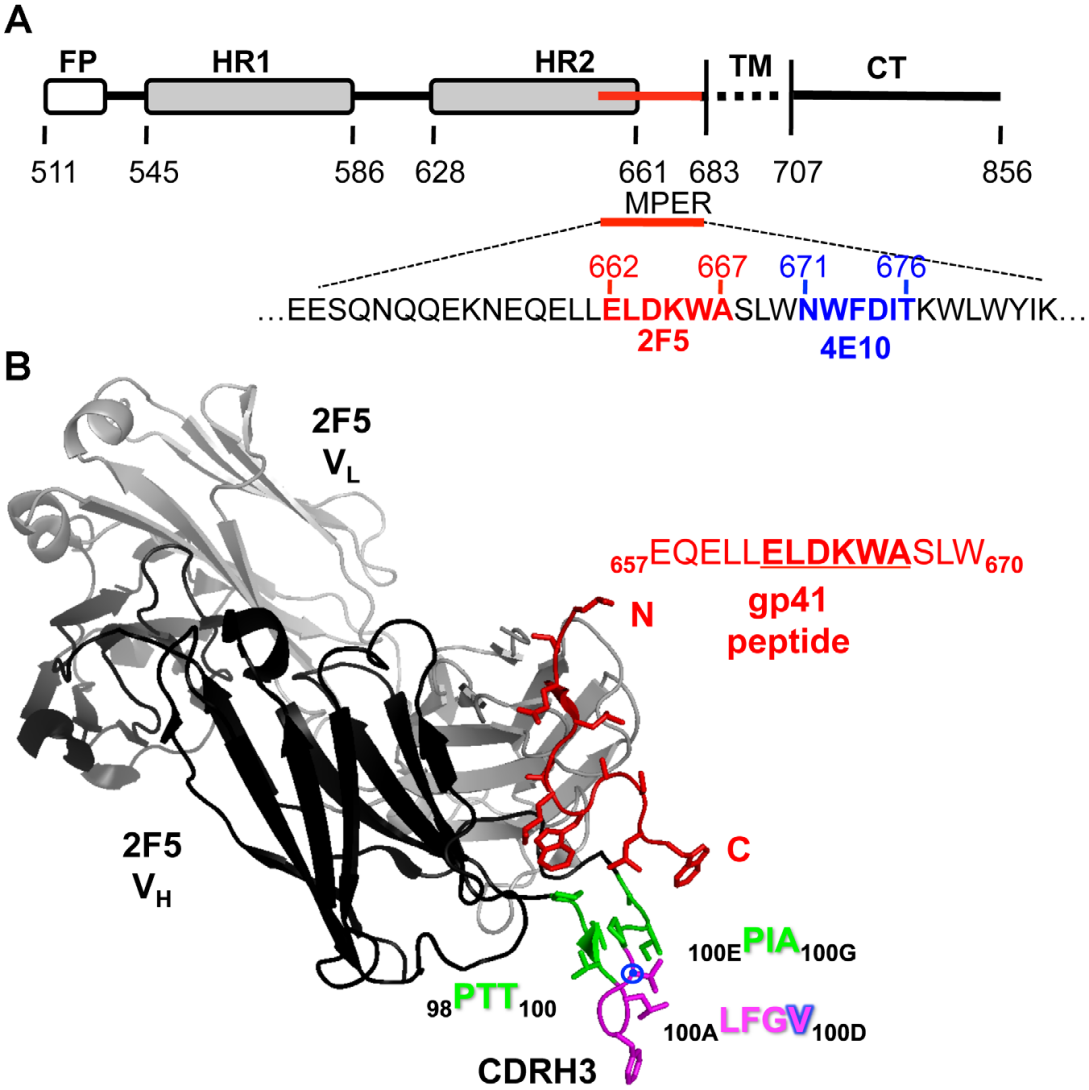
HIV-1 envelope glycoprotein gp41 schematic and structure of the 2F5 antibody-peptide complex. (A) The 2F5 (red) and 4E10 (blue) contiguous epitopes lie within the membrane proximal external region (MPER) of the HIV transmembrane envelope glycoprotein, gp41. (B) Crystal structure (PDB: 1TJI) of the antibody 2F5 heavy chain (black) and light chain (gray) in complex with the gp41 peptide (red). The third complementarity-determining region of the 2F5 heavy chain (CDRH3) is colored in green and magenta. Residues colored green (_98_PTT_100_ and _100E_PIA_100G_) were removed to decrease the length of the CDRH3 loop. Insertions were also engineered into this region to elongate the length of the loop. Residues at the apex of the CDRH3 in magenta (_100A_LFGV_100D_) were not altered. The residue V_100D_ is marked by the blue circle as this residue is substituted for by a W in several of the 2F5 variant antibodies.

The membrane proximal external region (MPER) of the HIV-1 envelope transmembrane glycoprotein, gp41, is the target of the two broadly neutralizing monoclonal antibodies (Mabs) 2F5 and 4E10 [1], [2] (Figure 1A). Although the exact mechanism of neutralization of these two antibodies is not yet determined, it is likely that they interfere with the process of fusion of the viral-to-target cell membranes. Recent data from our group indicate that the epitopes of the MPER antibodies are not exposed on most HIV-1 primary isolates and become accessible after engagement of the viral spike with receptors on the target cell surface [3]. To bring about membrane fusion, trimeric gp41 has to undergo large conformational changes, beginning with a structurally undefined native state interacting with the non-covalently associated exterior envelope glycoprotein gp120, to an energetically favored six-helix bundle conformation. Briefly, cellular receptors interacting with gp120 induce conformational changes in both gp120 and gp41. These conformation changes lead to the insertion of the gp41 N-terminal fusion peptide into the target cell membrane, eventually permitting fusion of the two membranes as gp41 collapses into the six-helix bundle state, mediating entry of HIV genetic information into the target cell. To mediate this process, the envelope glycoproteins must possess an inherent capacity for conformational change, and consistent with this requirement, the MPER region is well recognized for its conformational diversity. Multiple structural studies have highlighted its tendency to adopt flexible helical secondary structures as free peptides in solution [4], [5], [6], [7], [8], in the context of oligomeric post-fusogenic and intermediate gp41 forms [9], [10], [11] and when bound to the Mab 4E10 [12] or the non-neutralizing Mab 13H11 [13]. In contrast, when bound to the 2F5 Mab [14], [15] or the non-neutralizing Mab 11F10 [16] the MPER adopts an extended loop conformation, again attesting to its conformational flexibility. The 2F5 Mab structure in complex with its cognate peptide reveals that the peptide is bound in a cleft between the heavy and light chains making no contacts with most of the unusually long 22 amino acids (aa) CDRH3 loop [14], [15] (Figure 1B). Extensive studies of the CDRH3 corroborate its importance for both binding and neutralization activities of the 2F5 Mab, especially the hydrophobic residues present at the apex of the loop [17], [18], [19]. However, the exact role of the 2F5 CDHR3 in regards to how this region precisely contributes to binding and neutralization mediated by the 2F5 Mab remains undefined. The 2F5 Mab affinity for its epitope is enhanced when the epitope is presented in a lipid context, suggesting that, perhaps, the highly hydrophobic CDRH3 makes contact with the viral membrane [14], [20], [21]. Indeed, it was recently demonstrated that alterations in hydrophobicity at the tip of the CDRH3 were directly correlated with 2F5-mediated neutralization capacity [19].

In the present study, to further investigate the role of the 2F5 CDRH3 in binding, putative membrane interactions and neutralization mechanisms, we first made structure-based alterations in the length of the CDRH3 and assessed their impact on Mab function. We then increased the hydrophobicity and aromatic character of the resulting mutant CDRH3s by targeted tryptophan (W) and tyrosine (Y) substitutions. We measured binding affinities of the mutant antibodies for selected gp41 epitope probes by bio-layer light interferometry and we assayed their neutralization activity against a panel of HIV-1 strains of varying sensitivity. We found that the antibody 2F5 CDRH3 loop can sustain elongations of up to 26 residues in length and reductions to 18 residues in length and still maintain nanomolar range binding affinity to its cognate epitope peptide and retain some neutralizing activity. Targeted W and Y substitutions of the length-altered CDRH3s generally enhanced both the mutant antibodies' binding affinities and their neutralization activity to levels approaching or exceeding those of wt 2F5. These data indicate that a more diverse range of 2F5-like antibodies might be elicited by MPER-based candidate immunogens and yet retain HIV-1 neutralization capacity. We found a direct correlation between the antibody affinity constant for the MPER peptide and the inhibitory concentration (IC_50_) of HIV-1 neutralization that suggests a new property of the 2F5 CDRH3 loop that may be additional to and independent of its suggested membrane interaction. From these data, we propose an alternative model in which the CDHR3 loop destabilizes the helical secondary structure of the MPER in the context of the functional Env spike, acting downstream of direct core epitope contacts, allowing the antibody to induce the extended loop peptide-bound conformation observed in the Mab-peptide crystal structure. Finally, we demonstrate a direct interaction of both the wild-type (wt) 2F5 CDRH3 and the truncated, W-containing variant CDRH3s with elements of the downstream 4E10 region by alanine scanning mutagenesis of the MPER peptide. These data have direct and important implications for using MPER residues outside of the 2F5 core epitope for attempts to elicit broadly neutralizing 2F5-like antibodies as part of broadly effective vaccine.

## Results

### Design of the CDRH3 length-altered mutant 2F5 antibodies

**Figure 2:**
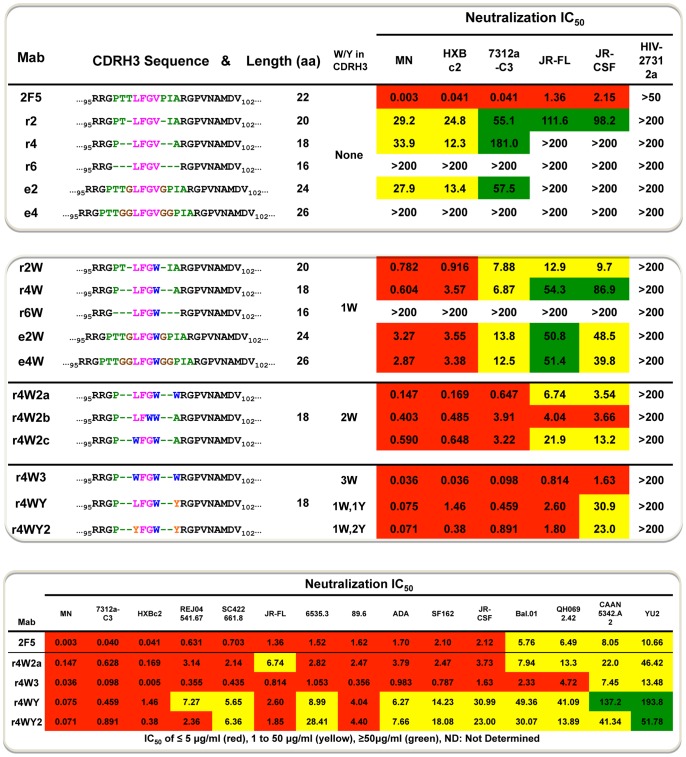
HIV neutralization values of the CDRH3 length-altered antibodies. The top and middle panels display the CDRH3 sequences of the length-altered CDRH3 2F5 variants and their HIV neutralization activities. Residues colored in magenta (LFGV) were conserved while flanking residues colored in green (PTT and PIA) were removed sequentially to produce the shorter loop antibodies. In brown are the glycine (G) residues inserted to elongate the CDRH3 length. The W and Y substitutions in the altered-CDRH3 are colored in blue and orange, respectively. The bottom panel shows neutralization values achieved by selected CDRH3-altered 2F5 variants against a larger set of HIV isolates.

The crystal structure of the 2F5 antibody in complex with its gp41 peptide epitope reveals the peptide embedded in a cleft between the heavy and light chains of the antibody in an extended-loop conformation [14], [15]. The unusually long (22 aa) 2F5 CDRH3 minimally contacts the antibody-bound peptide, however, this region is critical for 2F5 binding and neutralization (Figure 1B). Our primary goal in the present study was to investigate the length requirements of the 2F5 CDRH3 in regards to these functional capacities, in part because antibodies requiring a long CDRH3 to bind and neutralize virus might be difficult to elicit by vaccination. Previous studies demonstrate that ablation of the CDRH3 results in the complete loss of neutralization capacity. Another study revealed the importance of the residues at the apex of the loop (_100A_LFGV_100D_) for 2F5 neutralizing activity [17], [18], [19]. Therefore, for the CDRH3 length alterations we left residues at the tip of the loop unaltered and modified only the residues adjacent to each side of this apex (_98_PTT_100_ and _100E_PIA_100G_) (Figure 1B and Figure 2, top panel). We then reduced or elongated the CDRH3 loop by removing or adding two residues at a time, one from each side of the conserved apex residues. Residues were removed or inserted in this balanced manner in an attempt to preserve the local secondary structure of the CDRH3 loop. Since the precise identification of the D gene segment that is used to comprise the 2F5 CDHR3 is uncertain, we did not make modifications based upon the potential genetic elements encoding this region of the Mab. The modifications that we did make resulted in variant CDRH3s with lengths ranging from 16 to 26 aa. We called the resulting mutant 2F5 antibodies r2, r4 and r6 for reductions of the length of the loop of 2, 4 and 6 residues, respectively. For the elongated CDRH3s, we added glycine residues, two at a time, at each side of the tip (GG_100A_LFGV_100D_GG) and we named the antibodies e2 and e4, for elongation of 2 and 4 residues, respectively (Figure 2, top panel). The variant antibodies were produced by co-transfection of 293F mammalian cells with plasmids encoding the mutated heavy chains and the wt 2F5 light chain as described in the Methods section.

### Selective recognition of the MPER probes suggests a new role for the 2F5 CDRH3

**Figure 3:**
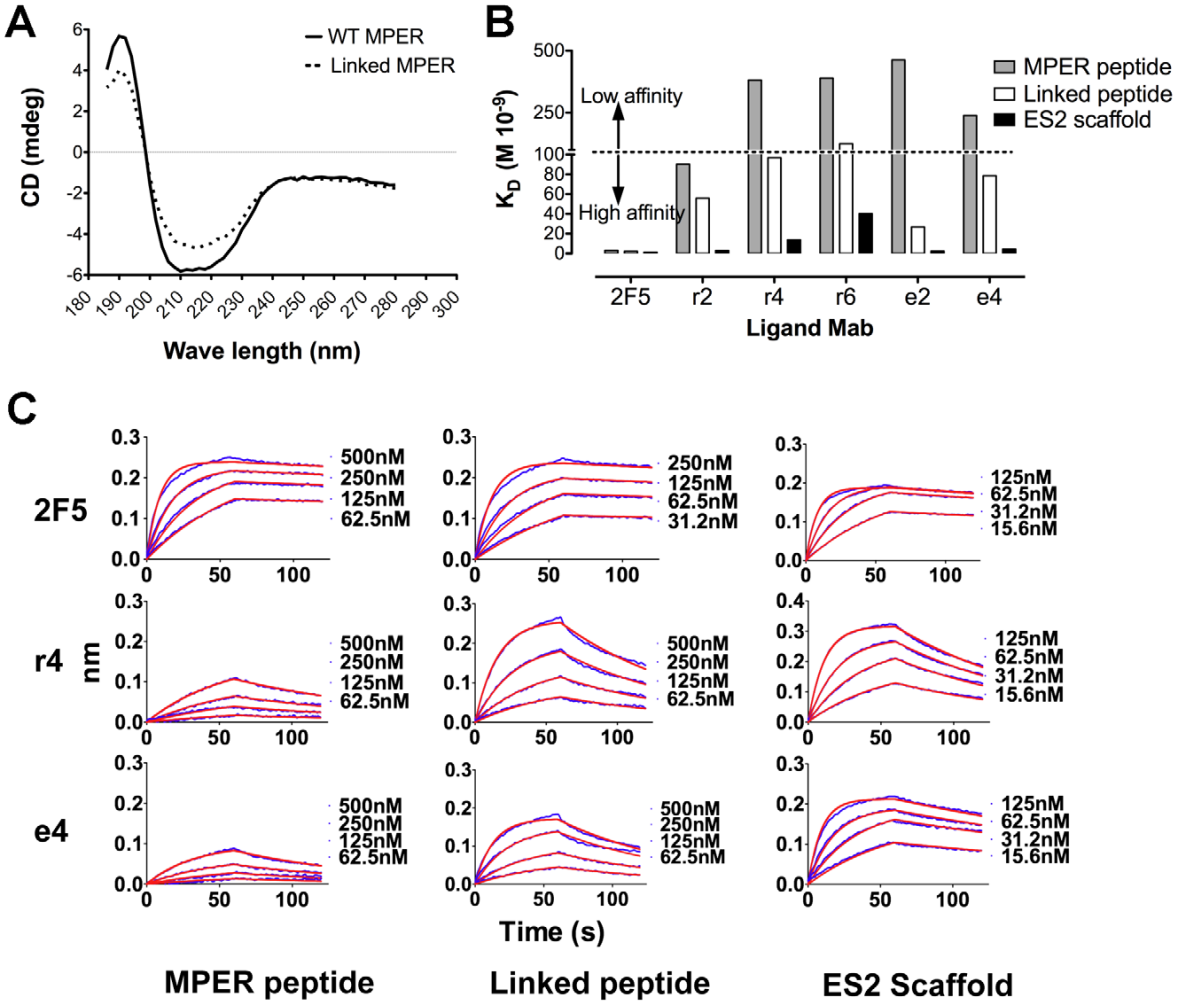
CD analysis and binding kinetics of the CDRH3 length-altered antibodies to selected MPER probes. (A) CD spectra of 10 µM wt MPER peptide and linked peptide in H_2_O. (B) Shown on the vertical axis are the affinity constants (K_D_), of the wt 2F5 Mab and the CDRH3 length-altered variants (r2, r4, r6, e2 and e4) for the selected MPER probes. Gray colored bars indicate antibody interactions with the MPER peptide analyte, white bars represent interactions with the linked peptide, and black bars indicate interactions with the ES2 2F5-epitope scaffold. The dotted line indicates an arbitrary cut off which segregates low affinity interactions (K_D_>100 nM) from high affinity interactions (K_D_<100 nM). For a complete description of binding kinetic constants see Table 1. (C) The Octet binding curves of wt 2F5 Mab to the three probes in solution are shown for the wt MPER peptide, the linked peptide, and the ES2 2F5-epitope scaffold. The experimental binding curves are shown in blue and the applied Languir 1∶1 model fitting of the curves are shown in red. Representative binding curves of the CDRH3-length altered 2F5 variants r4 and e4 are shown below. For the binding curves of all variants, see supplementary figures 1S and 2S.

To characterize binding kinetics of wt 2F5 and the CDRH3 length-altered 2F5 variant antibodies, we performed a bio-layer light interferometry kinetic binding analysis (Octet), similar in principle to the widely used surface plasmon resonance technology. We immobilized the immunoglobulin molecules on an anti-human IgG Fc-specific sensor and analyzed the MPER probes as free analyte in aqueous solution, completely free of a lipid bilayer. Based on their differential propensity to present the 2F5 epitope in a helical conformation, or not, three variant MPER probes were selected for this analysis. One version was an MPER peptide comprised of the complete wt sequence, encompassing both the 2F5 epitope and the contiguous 4E10 epitope, and containing a poly-lysine tail at the C-terminus to increase solubility [4], [5], [6], [7]. A second version of the MPER peptide that we refer to as “linked peptide” was generated to contain a poly-glycine linker separating the 2F5 epitope from the 4E10 epitope (Table 1). We chose to insert glycine residues to potentially disrupt the continuous helical conformation spanning from the 2F5 epitope to the 4E10 epitope observed in the MPER peptide under several crystallographic and NMR conditions. Glycine residues have high conformational flexibility and thus have poor helix-forming propensity due to entropic penalties. The circular dichroism (CD) spectra for both the wt MPER peptide and the linked peptide were consistent with a high content of alpha-helix, displaying positive bands at 192 nm and negative bands at 208 nm and 222 nm. The values for the negative bands were weaker for the linked peptide containing the glycine insert, suggesting, as expected, a lower alpha-helical content for this peptide compared to the wt MPER peptide (Figure 3A). Finally, we assessed binding in the ES2 2F5-epitope scaffold context which presents the 2F5 epitope exclusively in the antibody-bound, extended-loop conformation. This latter state is confirmed by crystallography [16].

**Table 1:** Antibody binding kinetic constants to selected MPER probes.

	MPER peptide^a^			Linked peptide^b^			ES2 scaffold^c^		
Ligand	k_on_ d	k_off_ e	K_D_ f	k_on_	k_off_	K_D_	k_on_	k_off_	K_D_
**wt 2F5**	2.3	0.7	**3.1**	3.6	0.7	**2.2**	10.1	1.3	**1.3**
**r2**	0.5	5.1	**90.2**	1.5	8.5	**55.8**	8.7	2.6	**3.0**
**r4**	0.2	8.1	**382**	1.0	10.5	**96.8**	6.6	9.3	**13.9**
**r6**	0.4	18.9	**390**	1.8	23.7	**125**	7.9	31.9	**40.2**
**e2**	0.1	8.7	**463**	1.3	3.5	**26.9**	7.4	1.7	**2.3**
**e4**	0.4	10.1	**239**	1.3	10.4	**78.6**	8.5	3.6	**4.2**

a **MPER peptide**: EQELL**ELDKWA**SLWNWFDITKWLWYIKKKKGSKKK.

b **Linked peptide**: EQELL**ELDKWA**SLGGGGSGGWNWFDITKWLWYIKKKKGSKKK.

c **ES2 scaffold**: scaffold protein displaying the 2F5 gp41 epitope stabilized in the antibody-bound conformation.

d Association rate (k_on)_ is measured in (1/Ms 10^5^).

e Dissociation rate (k_off_) is measured in (1/s 10^−3^).

f Affinity constant (KD) is measured in (M 10^−9^ or nM).

As expected, the wt 2F5 antibody displayed high affinity interactions with all three target analytes, with similar affinity constants (K_D_) in the 1 to 3 nM range (Table 1 and Figure 3B). In contrast, the CDRH3 length-altered antibodies displayed higher affinities for the ES2 scaffold protein than for either of the MPER peptides. Generally, the on-rate constant for the antibody recognition of the ES2 scaffold was 3- to 20-fold faster compared to antibody recognition of either of the other unconstrained MPER peptides (Table 1 and Figure 3C; For a complete set of binding curves see supplementary figures S1 and S2). The faster on-rate for ES2 is likely reflective of the fact that the variant 2F5 antibodies do not have to induce the extended-loop epitope conformation presented by this scaffold. Induction is likely necessary when starting with the helical conformation present in the unconstrained MPER peptides. Likely due to the impact of the conformational fixation, the highest affinity interactions of the mutant antibodies were observed for ES2 scaffold recognition. In these cases, the dissociation constants (K_D_) ranged from 2 to 40 nM, not much different from the values obtained for the wt 2F5 antibody. In contrast, the affinities for the MPER peptide were markedly lower, ranging from 90 nM to 460 nM. Slightly higher affinities were observed for the linked peptide, in an intermediate range from 26 nM to 125 nM (Table 1 and Figure 3B). These results are consistent with previous data that suggest that the antibody 2F5 CDRH3 plays a prominent role in the interaction with the MPER despite the fact that the antibody-peptide crystal structure reveals no contacts between most of the CDRH3 loop and the MPER peptide. This interaction likely happens before the antibody locks the 2F5 epitope into the extended loop conformation and is therefore not required when the Mabs interact with the ES2 scaffold that displays a preformed, extended-loop conformation, consistent with the faster on-rates in this context. Also note that the measurements presented here were performed in aqueous solution, indicating that a lipid environment is not required to detect such differences.

### Targeted W and Y substitutions recapitulate wt 2F5 neutralization activity

We next tested the antibodies capacity to neutralize HIV in a single-cycle infectivity assay using Env-pseudotyped virus and TZM-bl target cells. We selected a panel of viruses ranging from the highly sensitive HIV strains (HXBc2, MN and the HIV-2/HIV-1 MPER chimeric virus, 7312a-C3) to more resistant strains (JR-FL and JRCSF) and the HIV-2 strain 7312a as a negative control. The mutant 2F5 antibodies r2, r4 and e2 showed similar neutralization activity against the sensitive strains with 50% inhibitory concentrations (IC_50_) ranging from 12 µg/ml to 181 µg/ml, which were several orders of magnitude higher concentrations of the variant Mabs relative to those of wt 2F5. The variant 2F5 Mabs with the larger CDRH3 alterations, e4 and r6, displayed no neutralization activity against the strains tested. The 2F5 mutant antibody r2 was able to neutralize the more resistant HIV variants, but with values up to two orders of magnitude higher than those of the wt 2F5; ie, the r2 IC_50_ for JR-FL was 111 µg/ml and 98 µg/ml for JR-CSF whereas wt 2F5 values were 1.36 and 2.15, respectively (Figure 2, top panel). Given the weak neutralization activity displayed by the CDRH3 length-altered antibodies, we next investigated if a V_100D_W mutation, previously shown to increase the neutralization activity of the wt 2F5 antibody [19], would also imbue the same effect on the CDRH3 length-altered Mabs. We generated a second series of mutant antibodies by substituting the V_100D_ residue at the apex of the loop for a W and named the resulting antibodies r2W, r4W, r6W, e2W and e4W (Figure 2, middle panel). For example, r6W, denotes a reduction of 6 residues in the length of the CDRH3 and possessing the V_100D_W mutation. We then analyzed the antibody neutralization capacity against our original panel of pseudoviruses. The W substitution improved the neutralization activity for all variant 2F5 Mabs as much as 20-fold (Figure 2, middle panel). In the case of e4W, the added W was sufficient to convert the completely non-neutralizing antibody, e4, into a neutralizing antibody. Note that the V_100D_W substitution endowed the CDRH3 length-altered antibodies the capacity to neutralize the more resistant viruses of our panel, with the exception of r6W. That the antibody r6W did not exhibit any neutralization activity suggests that either the additional residues removed from the loop (P_98_ and A_100G_) are directly involved in neutralizing capacity or that the r6 CDRH3 was compromised in some indirect manner, perhaps by perturbation of local CDRH3 structural integrity.

**Figure 4:**
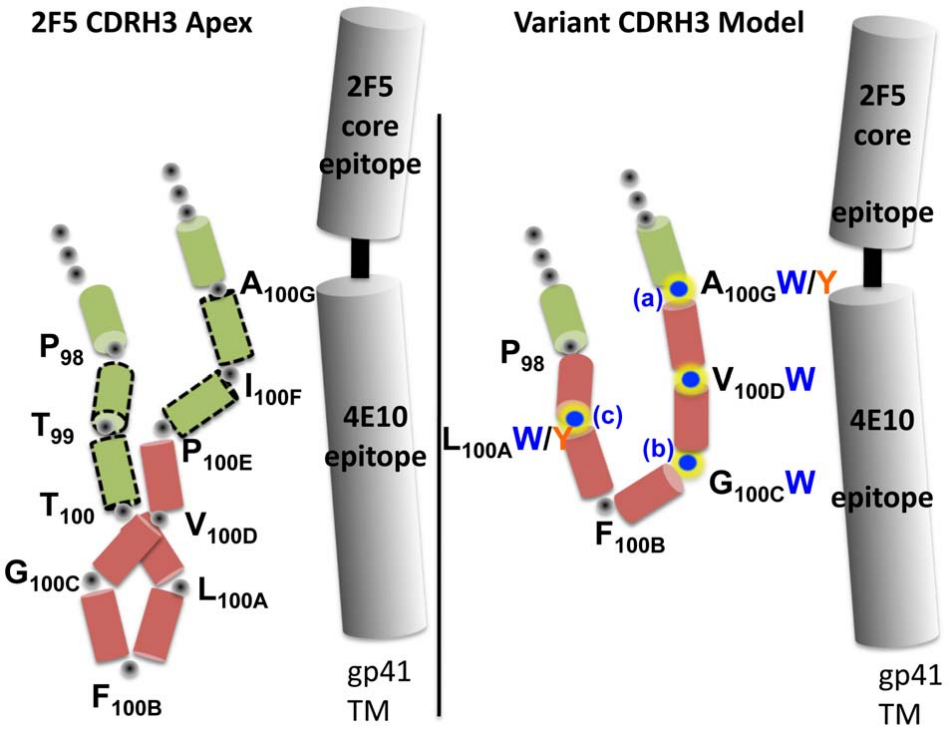
Schematic representation of the 2F5 wt and loop-shortened CDRH3 and approximate location of the MPER. Left, the wt 2F5 CDRH3 apex is shown as a schematic representation based upon the mAb structure. Residues P_98_ to A_100G_ are shown with each residue represented by a colored cylinder. On the right, a schematic of the length-altered version, where residues T_99_,T_100_, P_100E_ and I_100F_ , represented by the green cylinders with the discontinuous border, were deleted. The red residues, L_100A_, F_100B_, G_100C_ and V_100D_, were maintained for the length alterations as described in the text. The W and Y substitutions are noted next to the original residue in the variant schematic depiction (Rosetta modeling of a variant CDRH3 was performed, but clashes with peptide binding were assessed to not be compatible with the predicted conformations). The letters a, b and c represent the locations of the second W substitutions to generate antibody variants r4W2a, b and c, respectively.

Given that the single V_100D_W mutation demonstrated such a positive increase in neutralizing activity, we selected the loop-shortened r4W antibody, to introduce further W substitutions into the CDRH3. We added a second W substitution at three different positions within the apex of the loop: A_100G_W, G_100C_W and L_100A_W to generate antibodies r4W2a, r4W2b and r4W2c, respectively (Figure 2, middle panel and Figure 4). These W substitutions further increased the neutralization activity of these antibodies to levels just below the wt 2F5 antibody (Figure 2, middle panel). To potentially yet further increase neutralization activity, we selected r4W2a, our most potent mutant Mab, and added a third tryptophan at position L_100A_W, to generate r4W3 (Figure 2, middle panel and Figure 4). This additional W substitution fully reconstituted the neutralization activity of r4W3 relative to 2F5 wt against most viruses and, in most cases, r4W3 was slightly more potent than wt 2F5 (Figure 2 middle and bottom panels). The data indicated that the 2F5 CDRH3 can tolerate some plasticity in both terms of length and, as previously shown, hydrophobicity and yet still neutralize several representative HIV-1 isolates.

To better differentiate between either hydrophobicity or aromaticity as being critical for the activities of the CDRH3-shortened, W-containing 2F5 Mabs, we next changed two of the critical W residues to Y residues, since Y residues are still aromatic but less hydrophobic (Figure 2, middle panel and Figure 4). An additional consideration for these substitutions was the possibility that the W substitutions might imbue the CDRH3-shortened 2F5 Mabs with a hydrophobic nature and make them poly-reactive, replacement by the polar Y residue, which still possesses an aromatic ring in its side chain, might generate less poly-reactive 2F5 variants. Substitutions of two Y residues in place of the W residues resulted in a slight decrease in neutralization potency however this Mab variant still potently neutralized a broad panel of primary isolates displaying varying degrees of sensitivity to the 2F5 neutralizing Mab (r4WY2; Figure 2, bottom panel). These data suggest that the aromatic side chains of the substituted residues are contributing significantly to the binding energy responsible for neutralization activity of these length-altered antibodies; however, it appears that the bulkier and more hydrophobic side chain of the W residues better enhanced the neutralizing capacity of the mutant antibodies.

### Antibody neutralization correlates with the binding affinity to the MPER peptide

**Figure 5:**
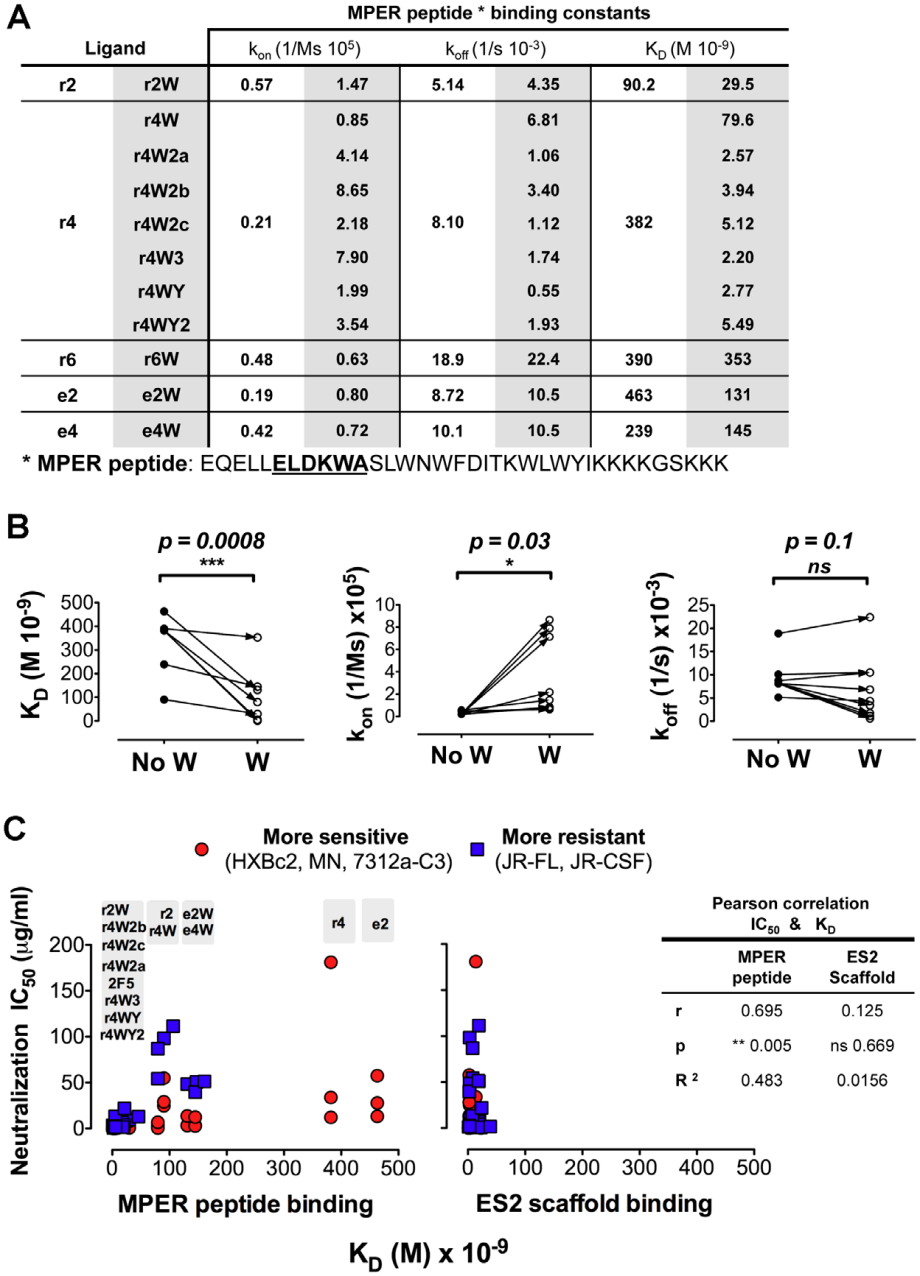
MPER Binding and HIV neutralization of mutant antibodies after W/Y substitutions. (A) Antibody affinity constants corresponding to antibody binding to MPER peptide before and after the W/Y substitutions, in white and shaded columns, respectively. (B) Statistical analysis comparing the means of the binding measurements to the MPER peptide from the antibodies before and after the W substitutions. The affinity constant (K_D_), the on-rate (k_on_) and the off-rate (k_off_) are shown on the vertical axis with each dot representing antibody binding to the MPER peptide. Closed circles represent antibodies with no Ws in the CDRH3 while open circles represent antibodies with W substitutions. Arrows indicate paired antibodies before and after the substitutions. The p values were obtained by subjecting the data to a paired t test statistical analysis. (C) Correlation between antibody K_D_ to MPER peptide, left panel, or K_D_ to ES2 scaffold, right panel, and HIV neutralization. Antibody IC_50_ values are shown on the vertical axis and the affinity constant (K_D_) to the MPER peptide in the horizontal axis. Red circles represent antibody IC_50_ values against the sensitive viruses (HXBc2, MN and 7312a-C3) and blue squares are IC_50_ against the more resistant isolates. Above the plots, in the gray boxes, are the names of the antibodies whose measurements are found directly underneath. The data were subjected to a Pearson correlation test, two-tailed resulting in a statistically significant p-value (p = 0.0052) and a positive correlation r-value (r = 0.695 ) for the wt MPER peptide and a non-significant p-value for the ES2 scaffold (p = 0.669 and r = 0.125).

**Figure 6:**
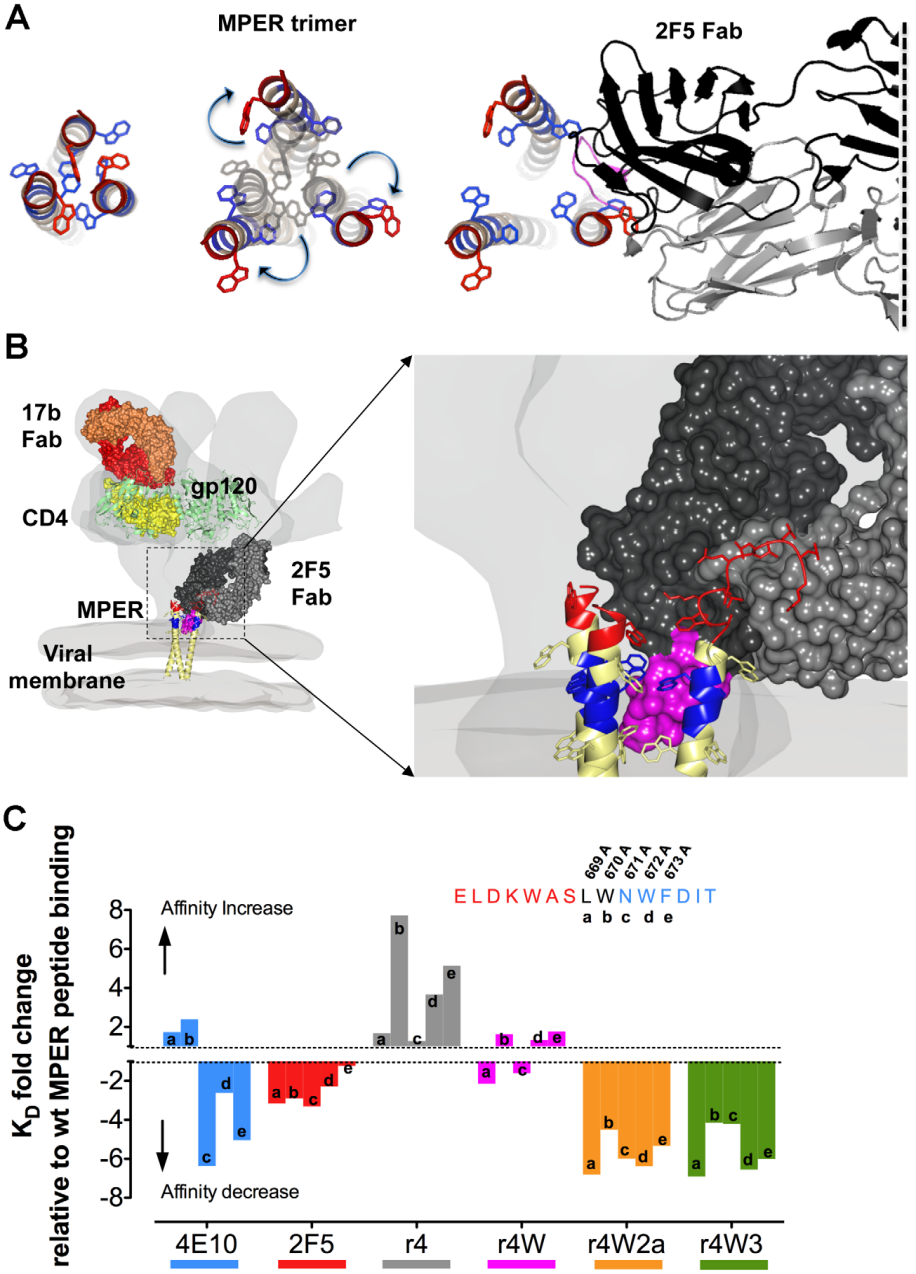
Model of the 2F5 antibody interacting with the HIV spike and MPER alanine scanning mutagenesis. (A) Left, top view of the trimeric MPER crystal structure (PDB: 3G9R) as it may appear in the HIV unliganded primary isolate trimer prior to receptor engagement. Middle, following receptor engagement there is a putative torsional movement of the individual protomers within the spike, exposing the MPER, as indicated by the blue arrows. Right, when the 2F5 antibody is finally bound to its epitope. (B) Model of the HIV spike with gp120 (green) bound to the antibody 17b (orange), the primary virus receptor CD4 (yellow) (PDB:2NXY) and the MPER (modified from PDB:3G9R) (red [2F5 eptitope], blue [4E10 epitope] and yellow) bound to the antibody 2F5 (black) with the wt CDRH3 (magenta) positioned within the groove between two of the MPER helices. The aromatic residues of the MPER are shown. (C) Alanine scanning of the 4E10 MPER region 669–673 and effects on recognition of 4E10, wt 2F5 and the CDRH3 and W/Y variants are shown. The bars represent fold-increase or -decrease in the binding constant (K_D_) of the Mabs to the alanine-substituted peptides compared to K_D_s for the wt MPER peptide. A positive bar indicates an affinity increase and a negative bar indicates an affinity decrease. The bars are color-coded for each ligand Mab. For context, the MPER residues comprising the 2F5 and 4E10 epitopes are shown and the residues 669–673 scanned by the alanine substitutions are as indicated.

Since the targeted W and Y CDRH3 substitutions generally increased the HIV neutralization activity of the mutant antibodies, we sought to determine if this improvement was also reflected in their binding properties. We performed kinetic binding measurements on the modified antibodies using the MPER peptide as analyte, since, in our initial binding analysis, higher affinity for this probe was associated with neutralization activity (Table 1 and Figure 2, top panel). With the exception of r6W, all antibody mutants carrying one (r2W, r4W, e2W and e4W), two (r4W2a, r4W2b and r4W2c) or three (r4W3) W substitutions displayed incremental increases in binding affinity (K_D_) to the MPER peptide, relative to their counterparts lacking the tryptophan substitutions (Figure 5A and Figure S3 and S4). The comparison of the mean K_D_ values of the antibodies carrying W substitutions with the mean of their counterpart antibodies was statistically significant (p = 0.0008) when measured on a two-tailed paired t test (Figure 5B). Similar high binding affinities were observed for the variants containing the two W/Y substitutions (r4WY, r4WY2) (Figure 5A). The increases in binding affinities for the W-containing variants was mostly due to increases in the Mab on-rates of binding to the MPER peptide (p = 0.03), while there was not a statistically significant difference in the off-rates (p = 0.1) (Figure 5B). These data demonstrate that increasing the hydrophobic or aromatic character of the length-altered CDRH3 loop facilitates recognition of the helical MPER peptide. We interpret the data to mean that the enhanced binding of the MPER by the W or Y substitutions results mostly from an improvement in the recognition of the epitope (faster on-rate) rather than an improvement in the stabilization of the interaction (no difference in off-rate). Additionally, when we compared the MPER peptide binding data with the antibodies' neutralization function, we found a statistically significant correlation (Pearson r = 0.695, p = 0.005) between neutralization activity (measured as the IC_50_) and the ability of the antibodies to recognize the MPER peptide (measured as the K_D_). However, there was no correlation when comparing the antibodies' neutralizing capacity with their ability to recognize the ES2 scaffold (Figure 5C and Figure S5). Taken together, the data suggest a new mode of interaction of the 2F5 antibody with its gp41 epitope in which the highly hydrophobic and/or aromatic CDRH3 loop destabilizes the helical secondary structure of the MPER by interacting with MPER residues outside the 2F5 epitope core to allow other elements of the 2F5 antibody to then induce the extended loop antibody-bound conformation observed in the crystal structure (Figure 6). These data indicate that the hydrophobic character or aromatic nature of the length-altered CDRH3 loop facilitates recognition of the MPER either as a monomeric protomer of the functional trimer, or within the putative MPER triple helix, to mediate neutralization and, importantly, in the absence of lipids. The fact that both binding of the MPER peptide and HIV neutralization are enhanced by the W/Y substitutions suggests that perhaps pi stacking of the phenyl rings with the W/F side chains of the helical MPER (i.e. W670, W672, F673 or W678) might contribute substantially to this functional enhancement, along with some degree of hydrophobicity.

To examine if the wt 2F5 antibody might interact with residues downstream of its core epitope, we analyzed 2F5 binding to MPER peptides harboring L669A, W670A, N671A, W672A and F673A substitutions. These residues are immediately C-terminal of the ELKWAS 2F5 core epitope. To this alanine scanning analysis, we added the r4 mutant series (r4, r4W, r4W2a and r4W3) displaying shorter CDRH3 loops and neutralization activities ranging from the very weak neutralizer, r4, to the most potent neutralizing 2F5 variant, r4W3. We reasoned that, if antibody contacts are made C-terminal of the ELKWAS core epitope, the A substitutions might also affect the binding of the r4 family antibodies and perhaps reveal specific residues that permit these 2F5 variant to regain their neutralizing potency. As a control, we also included the Mab 4E10 whose core epitope is contained within the region scanned by the A substitutions and should be sensitive to the 671–673 changes. Consistent with direct contacts at these residues, we observed decreased 4E10 affinity of the Ala mutant peptides 671/672/673 (Figure 6C and Figure S6). The Mab 2F5 presented decreased binding affinities for the A-substituted peptides in positions 669 through 672 suggesting weak contacts with this region, also noted previously [22]. Consistent with effects observed for the wt 2F5 antibody, the more potent neutralizers r4W2a and r4W3, with two and three W residues in their CDRH3 respectively, showed decrease binding affinities at all positions of peptides included in this alanine scan, including F673A, for which 2F5 displayed no sensitivity. These data suggest that the HCDR3-truncated, but W-rich CDRH3s, likely make hydrophobic interactions with residues 669–673 to regain their potent neutralizing capacity. In contrast, for the weak neutralizer r4, the A substitutions had the opposite effect, especially when the bulkier W and F side chains of residues 670,672 and 673 were mutated to A, suggesting that perhaps these residues are important for the stabilization of the MPER in a conformation only accessible to neutralizing antibodies. The A substitutions had no substantial effect in the peptide binding of the moderately neutralizing antibody, r4W, with the exception of a small decrease in affinity at position 669 (Figure 6C and Figure S6).

### Alterations of the 2F5 CDRH3 affect antibody binding to cardiolipin (CL) and beta-2-glycoprotein I (B2G1)

**Figure 7:**
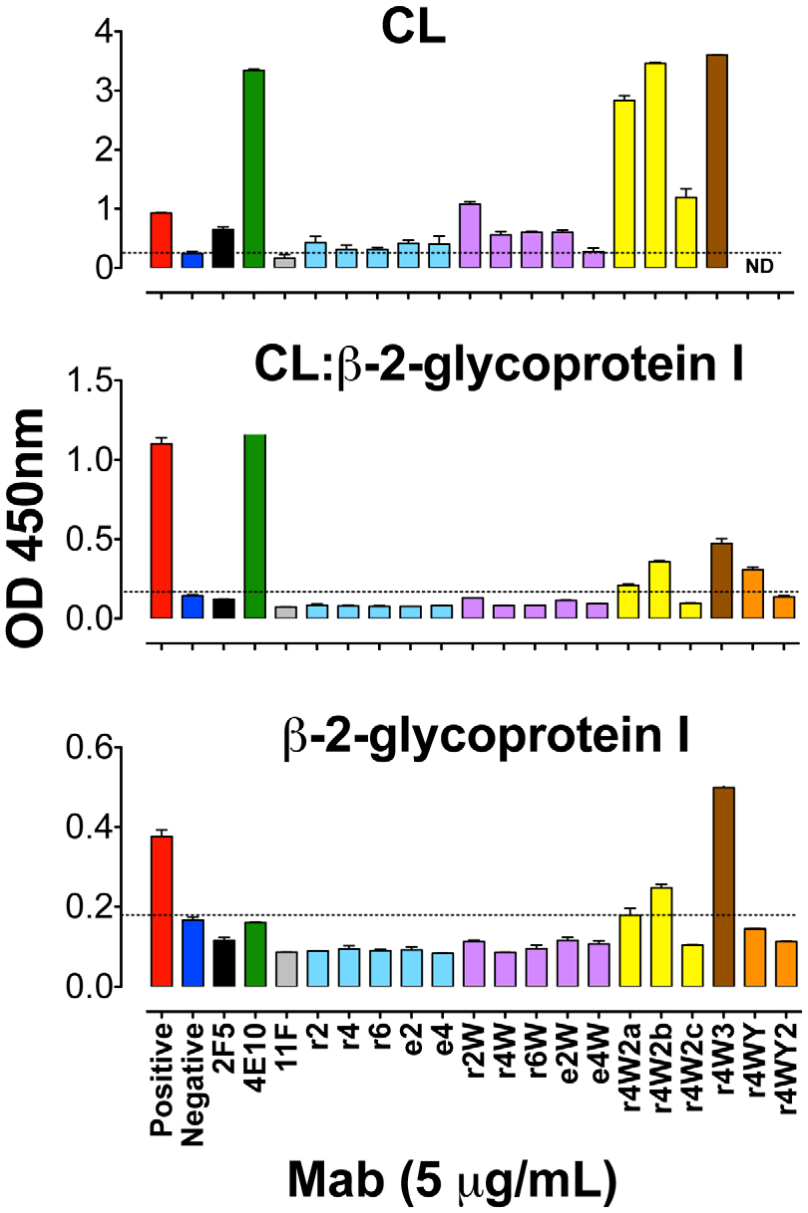
Antibody binding to CL:B2G1. Bars represent ELISA optical density readings at 450 nm wavelength corresponding to the binding of antibodies (2F5 in black, 4E10 in green, 11F10 in gray and the CDRH3-altered variants in blue, violet, yellow, brown and orange) to CL (top panel), CL:B2G1 complex (middle) and B2G1 (bottom). Light blue color designates CDRH3 altered antibodies with no W substitutions, violet with one W substitution, yellow with two W substitutions, brown with three W substitutions, and orange with one W and one or two Y substitutions. The red and dark blue bars represent the positive and negative binding levels of control IgG included in the assay. The dotted line indicates an arbitrary “cut-off” between the negative and positive values.

Both the Mabs 2F5, and especially 4E10, have been reported to be poly-specific, binding to a set of human molecules, including CL [23], [24], [25]. Here, we selected three binding assays to measure the effect that our multiple CDRH3 alterations might have in the poly-reactive nature of the resulting 2F5 variants. Based on a previous study [25], we selected two antibody concentrations (5 and 1 µg/ml) and assessed binding to CL alone, CL:B2G1 complexes and to B2G1 (also known as Apolipoprotein H), alone. Antibodies that recognize the CL:B2G1 are associated with pathogenic autoimmunity [26]. Consistent with the previous study assessing CL binding, at 5 µg/ml, both 2F5 and 4E10 bind to CL, with 4E10 displaying the strongest reactivity, whereas at 1 µg/ml the antibody 2F5 is negative for CL binding, while 4E10 remained positive (Figure 7 and Figure S7). In addition to 4E10 and 2F5, we also selected the non-neutralizing 11F10 mouse monoclonal antibody, a 2F5-epitope binding antibody that does not possess a long hydrophobic CDRH3 and does not react with CL [16], [27], [28]. The antibody 4E10 was positive at both concentrations in the CL alone and the CL:B2G1 complex assays, however, negative in the B2G1 assay. The antibody 2F5 reacted only with CL alone at the 5 µg/ml concentration and was not reactive with the CL:B2G1 complex or the B2G1 alone. 11F10 antibody was negative in all assays. Our original alterations in the length of the CDRH3, both reductions (r2, r4 and r6) and elongations (e2 and e4), resulted in antibodies that weakly bound CL at 5 µg/ml and generated a signal that was weaker than the binding observed for the antibody 2F5 (Figure 7). The mutant antibodies carrying one tryptophan substitution (r2W, r4W, r6W, e2W and e4W) reacted with CL at a level comparable with that of the 2F5 antibody, with the exception of e4W, which was non-reactive. Interestingly, the e4W antibody which exemplifies an MPER-directed neutralizing antibody, exhibited no reactivity to CL or CL:B2G1. The tryptophan substitutions, two or three, (r4W2a, r4W2b, r4W2c and r4W3) strongly enhanced CL reactivity, surpassing the levels of the wt 2F5 antibody and matching that of the Mab 4E10 (Figure 7 and Figure S6), but were generally lower on CL:B2G1 complexes. Generally, it appears that the antibodies that displayed stronger binding to CL also possessed higher neutralization activity. The data suggest that the antibody 2F5 CDRH3 loop, and its hydrophobic or aromatic character, is responsible for the relatively weak CL binding, and, perhaps as well, its poly-reactive nature. Finally, the Y-substituted r4WY2 showed no reactivity to CL:B2G1 nor B2G1 at the 5 µg/ml, suggesting that MPER directed neutralizing antibodies may not possess the signature of bona fide auto-reactive Mabs while maintaining HIV neutralization activity.

## Discussion

The binding sites of HIV-1 broadly neutralizing antibodies (bnAbs) reveal sites of accessibility on the viral spike and present pathways for immunogen design to penetrate evolved barriers that successfully exclude access by most host-generated antibodies. Sequence analysis and structural characterization of these bnAbs reveal that several of these bnAbs possess unusually long and often protruding CDRH3s [12], [14], [29], [30], [31]. Antibodies with such long CDRH3 loop lengths might be difficult to elicit via vaccination, based in part on their relative infrequency due to limits imposed by D gene starting sequences and N nucleotide addition, but also due to possible counter selection imposed by self-reactivity encountered during transitional B cell stages from the bone marrow to the peripheral naïve repertoire. Generally, the CDRH3 is intimately involved in the binding to antigen and often modifications in this loop result in loss of binding and, relevant to this study, neutralization capacity. In the case of the antibody 2F5, the apex of CDRH3 does not directly contact the gp41 peptide epitope [14], [15], however, this CDRH3 region is essential for neutralization [17], [18], [19]. Binding and neutralization of HIV primary isolates by 2F5 likely occur following receptor engagement which exposes the MPER, but under certain conditions the spike can be inactivated in a receptor-independent manner, resulting in a slow dissociation of gp120 from gp41 [32]. Viral accessory proteins, such as Nef, also affect MPER accessibility and 2F5/410 neutralization potency [33].

In this study, we generated 2F5 Mab CDRH3 variants with both shorter and longer CDRH3 loops that still displayed a range of binding and neutralization activities. When these CDR loop length alterations were combined with aromatic residues on the flanks of the CDRH3, high affinity binding, in the absence of lipid, and neutralization activity equivalent to that of wt 2F5 was observed. These data suggest that the 2F5 CDRH3 interacts with aromatic MPER side chains outside of the 2F5 core epitope. That loop shortening still permits such aromatic residue-dependent interactions, and that that binding effects to MPER peptide can be observed in aqueous solution, argue against the model proposed previously, and recently, that the long 2F5 CDRH3 dips into the viral membrane to extract its epitope [14], [21]. In addition, the CDRH3 analysis presented here better defines the range of antibodies targeting the 2F5 gp41 MPER that might be able to neutralize HIV-1.

Specifically, we show that variations in the length of the CDRH3 can be tolerated in terms of high affinity binding and neutralization equivalent to wt, provided that the W and Y residues are present at the CDRH3 apex. The W substitutions generally increased the antibody neutralizing capacity of all mutants except for the shortest r6W CDRH3, suggesting that hydrophobic character or the aromatic nature of the W residues may play a more prominent role than its length in regards to neutralization. Encouragingly, Y residues could also restore neutralizing activity of CDRH3-shortened 2F5 variants, increasing the range of antibodies that, if elicited, might still efficiently recognize the HIV-1 MPER. Restoration of binding and neutralization to wt 2F5 levels could be accomplished by targeted substitutions of aromatic residues on the flanks of the CDRH3 apex. Variability in both the 2F5 CDRH3 length and sequence broadens the spectrum of antibodies targeting the 2F5 epitope that can be permitted, revealing the possibility that antibodies elicited via vaccination, displaying more frequently generated short CDRH3 loops, can effectively function as neutralizing antibodies to this region.

Although much is known about the 2F5 antibody, its mode of interaction with the HIV-1 Env spike, such as putative CDRH3 contacts with the viral membrane or as suggested here, within the HIV-1 trimeric spike itself, are not yet fully elucidated. Our alterations within the 2F5 CDRH3 showed that interactions with peptide were facilitated by the W residues substituted in the length-altered CDHR3 loops and that such modifications greatly enhanced Mab neutralization activity. Aromatic residues such as W and Y are overrepresented in antibody-combining sites, perhaps due to evolutionary selection. Biochemically this might be advantageous to imbue the Mab a capacity to stack their aromatic side chains, producing cooperative binding effects to antigen [34], [35], [36]. Hydrophobic interactions can account for large contributions to binding energies in protein-protein interactions and for 2F5, hydrophobicity of the CDRH3 appears critical for neutralization. The lack of a hydrophobic CDRH3 might in part account why antibodies generated to the structurally defined 2F5 epitope-peptide alone, such as the 11F10 antibody, lack neutralization activity despite displaying high-affinity binding to the 2F5-epitope-peptide [16], [27]. In the case of the CDRH3-altered Mabs presented here, the neutralization-enhancing W substitutions, which also increased antibody affinity for the MPER peptide, perhaps by interacting with MPER hydrophobic/aromatic residues immediately downstream of the 2F5 core epitope into the 4E10 epitope region (residues 669–673, see Figure 4 and Figure 6). This model is supported by the alanine scanning binding analysis performed in the 669–673 region of the MPER peptide. We would suggest that for wt 2F5, weaker contacts are made with these MPER residues as well, and perhaps even further downstream due to its longer CDRH3 length. Consistent with this model, a published crystal structure of a trimeric MPER construct reveals a parallel triple-stranded coiled held together by a stabilizing hydrophobic core, composed mostly of aromatic residues, in which the epitopes of antibodies 2F5 and 4E10 are occluded from antibody recognition of this conformation [10]. Recent cryo-electron microscopy studies show that both envelope glycoproteins gp120 and gp41 experience torsional movements after engagement of the viral receptor and co-receptor [37], [38], which may then expose the 2F5 and 4E10 epitopes for antibody recognition by these neutralizing Mabs [3]. Considering the structural information provided in the aforementioned studies, and the mutagenic, binding and neutralization data presented here, it is plausible to speculate that the 2F5 CDRH3 may interact with the hydrophobic residues located within a single protomer of the MPER or in between the MPER helices in the hydrophobic interior stalk of the trimeric spike. This positioning of the CDRH3 loop would permit the substituted W residues to interact with other aromatic residues abundant in the MPER, generating π-stacking interactions to confer additional binding energy. Curiously, the positioning of the second W in the CDRH3 loop (as in antibodies r4W2a-b-c) had an impact in their neutralization activity; namely, the A_100G_W and G_100C_W were more favorable than the L_100A_W substitution. By inspection of the crystal structure of the 2F5 antibody in complex with its cognate peptide epitope, one can envision that the side chains of W residues at positions A_100G_ and G_100C_ would lay orthogonally and adjacent to the main chain axis of the 2F5-bound peptide, positioning the loop residues C-terminal of the 2F5 epitope and proximal to the 4E10 epitope.

The binding kinetic analysis presented here showed a statistically significant correlation between the antibody binding affinity for the MPER peptide, driven mainly by an increase on the on-rate constant, and the HIV-1 neutralization activity of the antibody as determined by its IC_50_ value. That is, the faster is the on-rate of the antibody for the peptide, the more potent the antibody is in regards to HIV-1 neutralization assay. Taken together, we postulate that the antibody 2F5 CDRH3 loop interacts with the helical MPER, disturbing its helicity and allowing the antibody to induce the extended loop peptide-bound conformation into the Mab cleft observed in the antibody-peptide complex crystal structure. It also appears that interaction with the MPER by the CDRH3 mutants and wt 2F5 is downstream of the 2F5 core epitope and implies that more of the MPER should be included in vaccine candidates designed to re-elicit 2F5-like antibodies. The model that the MPER has helical propensity does give rise to the question of how was 2F5 initially elicited? We would suggest that, following receptor engagement, this region has evolved to undergo the conformational changes required for fusion and entry and that it has a natural tendency to sample multiple conformations during this process, both helical and beta turn. We would speculate that the B cell displaying 2F5 as its B cell receptor may have trapped the beta turn conformation, and the soluble antibody that we study has the property of being able to extract this conformation from the native Env, an apparently rare property for antibodies elicited against the MPER.

The CL binding assay showed that some variant Mabs displaying the highest neutralization activity, possessing the W substitutions, also presented the strongest reactivity to CL but not to B2GI. Recognition of B2GI itself or to CL-B2GI complexes and less so to CL alone, is a pattern that is associated with pathogenic autoreactive antibodies. Since most the variant Mabs possessing the Ws, despite binding to CL, do not well recognize the complex, concerns that these antibodies might be auto-reactive and be deleted from the naïve repertoire are reduced. Encouragingly, we demonstrate that Y residues in lieu of W substitutions could be accommodated in the loop length-altered antibodies and diminished or abolished CL reactivity with minimal effects in neutralization capacity.

In sum total, the data presented here provide hope for vaccine efforts to re-elicit 2F5-like Mabs since our CDRH3 modifications on the 2F5 antibody illustrate that many 2F5-related variants can still bind the MPER and, most importantly, potently neutralize HIV-1 primary isolates.

## Materials and Methods

### Proteins and peptides

The ES2 protein was expressed in 293F cells and purified from filtered supernatant by affinity chromatography using the wt 2F5 Mab. MPER peptides were synthesized with a poly-lysine tail at the C-terminus to facilitate solubility, Genscript (Piscataway, NJ). The plasmids encoding the wt 2F5 Mab were generated by *de novo* synthesis (Genscript) of the heavy and light chain genes and subsequently cloned into the CMVR mammalian expression vector. The Mabs containing the length-altered CDRH3 loops were generated from the wt 2F5 heavy chain plasmid by site-directed mutagenesis. The primers for the site-directed mutagenesis PCR reactions were synthesized by IDT (Integrated DNA Technologies, Coralville IA).

### Expression and purification of antibodies

All antibodies were generated by co-transfecting 293F cells with a unique mutant heavy chain and the 2F5 wt light chain encoding plasmids, at a 3∶2 ratio. Supernatants were collected 5 days after transfection, filtered, and run over a protein A chromatography column to purify the immunoglobulin molecules from the supernatant. The isolated antibodies were buffered exchanged to PBS pH 7.4 using the Amicon (30 kD) ultra-centrifugation filtering device from Millipore and stored at −80°C.

### Circular dichroism (CD) spectroscopy

CD measurements were performed using an Aviv model 420 spectrometer. Measurements were recorded at 25°C at 2 nm/min scan rate. The peptide concentration was 10 µM in H_2_O, and spectra were collected with a 0.1 cm path-length in the far-UV region (280 nm-185 nm).

### Bio-Layer interferometry binding assay

For Bio-layer interferometry, the Mabs were immobilized on anti-human IgG Fc biosensors (ForteBio, incorporated). The biosensors were hydrated in PBS pH 7.4, 0.2% Tween 20 for 10 minutes prior to starting the assay. Briefly, using an Octet Red instrument (ForteBio, incorporated) hydrated biosensors were immersed for 60 seconds at 1000 rpm in individual wells of a 96 well black microplate (Greiner bio-one) containing 200 µL of solution with a concentration of 10 µg/mL of antibody in PBS pH 7.4, 0.2% Tween 20. After a 60 second wash in PBS pH 7.4, 0.2% Tween 20 at 1000 rpm, bio-sensors with captured antibody were immersed in the analyte-containing well for 60 seconds at 1000 rpm to allow association of ligand and analyte. Analyte concentrations were two-fold serially diluted within the following range of concentrations 500 nM down to 7.81 nM. The analytes used were: the 2F5-epitope-scaffold ES2 protein, a wt MPER peptide (EQELLELDKWASLWNWFDITKWLWYIKKKKGSKKK) and a MPER linker peptide (EQELLELDKWASLGGGGSGGWNWFDITKWLWYIKKKKGSKKK) where the 2F5 epitope is separated from the 4E10 epitope by non-helix forming multi-glycine linker. Dissociations were allowed for 60 seconds at 1000 rpm by immersing complexes on the biosensor onto wells containing PBS pH 7.4, 0.2% Tween 20.

### Alanine scanning of the MPER peptides

Six peptides of the same length (35aa) were used for this alanine scanning analysis with sequences based on the wt MPER peptide used in the light interferometry experiment (EQELLELDKWAS_669_LWNWF_673_DITKWLWYIKKKKGSKKK). Residues 669–673, located C-terminal of the 2F5 epitope core ELDKWAS, were individually mutated to alanine. The binding measurements were done using light interferometry (ForteBio) where the antibodies 2F5, 4E10, r4, r4W, r4W2a and r4W3 were immobilized on the tip sensor and the peptides were in aqueous solution. The measurements were carried out using specifications detailed in the prior section. Binding affinity constants (K_D_, k_on_ and k_off_) were calculated for all antibodies with the Ala-substituted peptides and the wt MPER peptide. We then calculated fold-changes of these parameters for each antibody and plotted the fold-increase or fold-decrease in K_D_ as a positive or negative bar, respectively.

### Neutralization assay

The assay was performed in a 96-well microtiter plate with 20,000 TZM-bl cells distributed into each well. Titration of the Mabs were done in a separate plate and pre-incubated with virus for 1 hour at 37°C, then added to the target cells. Approximately 48 hours after addition of virus to the target cells to allow infection, the cells were lysed, and RLU were measured using white solid OptiPlates-96F plates (PerkinElmer, Boston, MA) and a Veritas Luminometer (model 1420-061; PerkinElmer) that injects luciferase assay substrate (Promega) into each well. Pseudoviruses were prepared by cotransfecting 293T cells with an Env expression plasmid containing a full-length gp160 *env* gene and an *env*-deficient HIV-1 backbone vector (pSG3ΔEnv). Pseudotyped virus-containing culture supernatants were harvested two days after transfection and stored at −80°C. For neutralization assays, each pseudotyped virus stock was diluted to a level that produced approximately 100,000 to 500,000 RLU.

### CL and beta-2-glycoprotein I binding assays

The CL binding assay was carried out in an ELISA format with a kit purchased from Alpha Diagnostic International, TX (CL) and Genesis Diagnostics, NH (CL+ß-2-glycoprotein I complex and ß-2-glycoprotein I). All experimental antibodies were diluted to either 1 µg/mL or 5 µg/mL utilizing a provided diluent containing bovine serum antigen (BSA) and goat serum to reduce background non-specific binding. Samples were run in duplicate at both antibody concentrations in a 96 well plate coated with purified CL antigen, or CL saturated with beta-2-glycoprotein I, or beta-2glycoprotein alone. After one hour incubation at RT, the antibodies were washed five times to remove unbound antibody. Secondary anti-human IgG Fc HRP-conjugated secondary antibodies were allowed to bind for 30 minutes, followed by five washes. Then, chromogenic substrate (TMB) was added and color was allowed to develop for 10 minutes. The reaction was terminated by adding a stopping solution (0.25 M H_2_SO_4_) and absorbance was then measured at 450 nm.

### Statistical analysis

Statistical analyses were performed using GraphPad Prism version 5.0 (GraphPad Softwared Inc.). For the comparison of the antibodies' binding kinetic parameters to the MPER peptide (K_D_, k_off_ and k_on_) in Figure 3A, before and after the substitution of W residues, we used a paired t test. The data passed a D'Agostino & Pearson omnibus normality test. The alpha tests level was set a 0.05, where n = 9 pairs and the tests were two-tailed. For the K_D_ comparison the p = 0.0008 and the R^2^ was 0.7714 , for the kon comparison the p = 0.0308 and the R^2^ was 0.4611, and for the koff comparison the p = 0.1094 and the R^2^ = 0.2885.

To determine if the Antibody Inhibitory Concentration (IC50 µg/mL) correlated with the antibody binding to the MPER peptide measured as K_D_ values as in Figure 3C, we applied a two-tailed Pearson correlation analysis to both sets of experimentally determined values. The results of this test show a Pearson r = 0.695, a p-value = 0.0052 and R^2^ = 0.483.

## Supporting Information

Figure S1 **Octet binding curves of reduced CDRH3 length antibodies to selected MPER analytes.** Displayed on top of each graph are the name of the ligand Mab and the corresponding MPER analyte. In blue are the experimental curves and in red the curves corresponding to the applied Langmuir 1∶1 model fit. The analyte concentrations corresponding to the curve series are shown to the right of each graph in namolar units.
(TIF)

Figure S2 **Octet binding curves of elongated CDRH3 antibodies to selected MPER analytes.** Displayed on top of each graph are the name of the ligand Mab and the corresponding MPER analyte. In blue are the experimental curves and in red the curves corresponding to the applied Langmuir 1∶1 model fit. The analyte concentrations corresponding to the curve series are shown to the right of each graph in namolar units.
(TIF)

Figure S3 **Effect of W substitutions on binding to the MPER peptide by reduced CDRH3 length antibodies.** The graphs are organized in pairs to show the effects of the W substitution on the antibody binding to the MPER peptide before (left) and after the W substitution (right). In blue are the experimental curves and in red the curves corresponding to the applied Langmuir 1∶1 model fit. The analyte concentrations corresponding to the curve series are shown to the right of each graph in namolar units.
(TIF)

Figure S4 **Effect of W substitutions on binding to the MPER peptide by elongated CDRH3 antibodies.** The graphs are organized in pairs to show the effects of the W substitution on the antibody binding to the MPER peptide before (left) and after the W substitution (right). In blue are the experimental curves and in red the curves corresponding to the applied Langmuir 1∶1 model fit. The analyte concentrations corresponding to the curve series are shown to the right of each graph in namolar units.
(TIF)

Figure S5 **Binding affinity constants to wt MPER peptide and ES2 scaffold and Mab neutralization IC_50_s.** The table displays the values used to calculate the correlation between binding and neutralization in Figure 5. Mab affinities (K_D_) to the wt MPER peptide and the ES2 scaffolds are shown along side with the IC50s for a panel of five pseudoviruses.
(TIF)

Figure S6 **Binding kinetic constants to alanine substituted MPER peptides.** Shown in this table are the affinity constant (K_D_), the on-rate (k_on_) and off-rate (k_off_) for the Mabs used in the alanine scanning analysis. Next to each parameter is the corresponding fold-change with respect to antibody binding to wt MPER peptide. A red/negative number represents an unfavorable change (i.e.: decrease in affinity or decrease in on-rate or a faster off-rate) whereas a blue/positive number represents a favorable change.
(TIF)

Figure S7 **Antibody binding to CL:beta-2-glycoprotein I.** Bars represent ELISA optical density readings at 450 nm wavelength corresponding to binding of antibodies (2F5 in black, 4E10 in green, 11F in gray and the CDRH3 altered variants in blue, violet, yellow, brown and purple) at 1 µg/mL concentration to CL (top panel), CL:beta-2glycoprotein I complex (middle) and beta-2glycoprotein I (bottom). Blue color designates CDRH3 altered antibodies with no W substitutions, violet with one W substitution, yellow with two W substitutions, brown with three W substitutions, and purple with one W and one or two Y substitutions.
(TIF)
