# Supplementary figures and images for: Structure-guided Alterations of the gp41-directed HIV-1 Broadly Neutralizing Antibody 2F5 Reveal New Properties Regarding its Neutralizing Function
Source: PLoS Pathog. 2012 Jul 19;8(7):e1002806. doi: 10.1371/journal.ppat.1002806 (PMC3400562; doi:10.1371/journal.ppat.1002806)

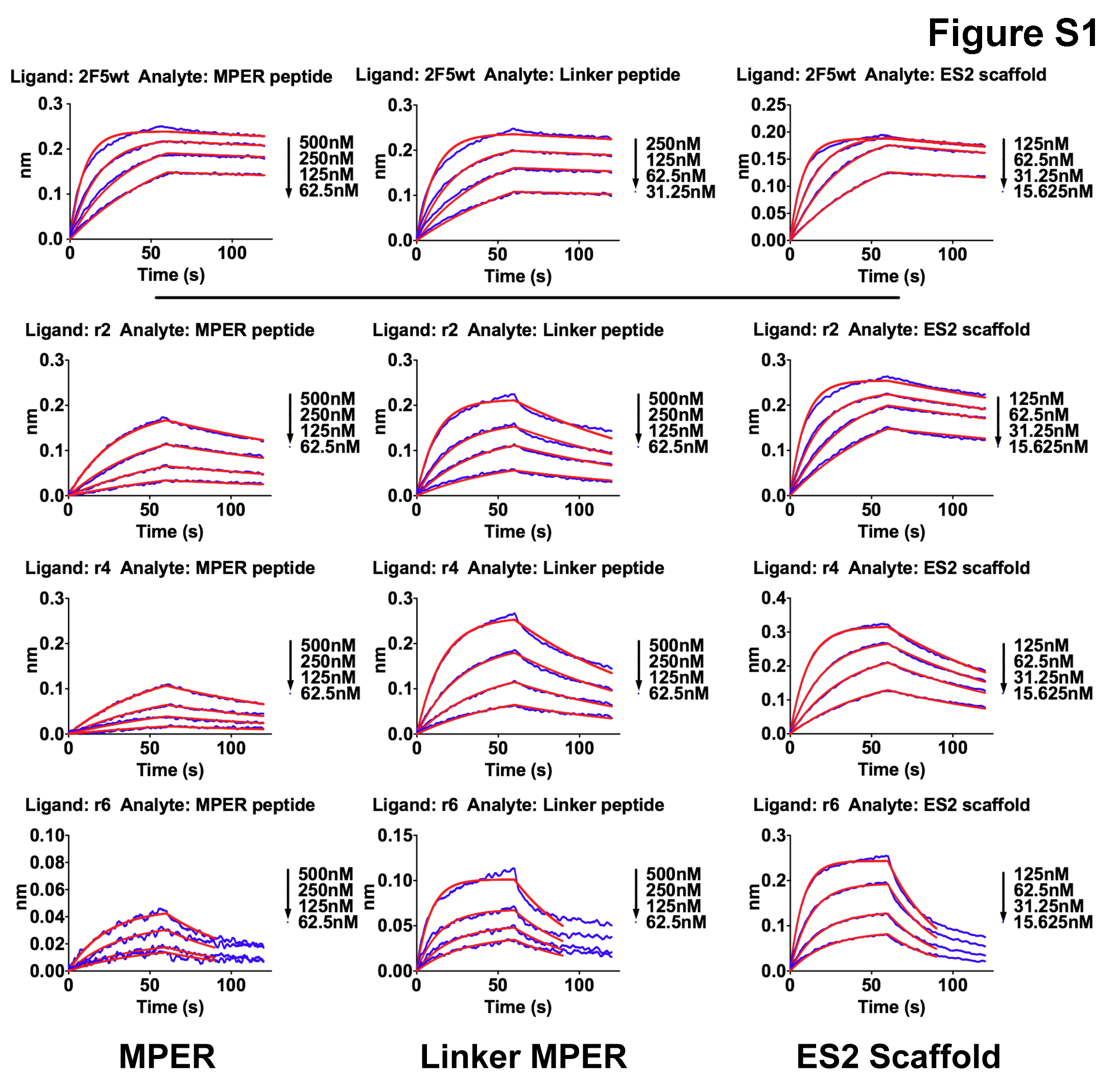

Supplement: Figure S1 — Octet binding curves of reduced CDRH3 length antibodies to selected MPER analytes. Displayed on top of each graph are the name of the ligand Mab and the corresponding MPER analyte. In blue are the experimental curves and in red the curves corresponding to the applied Langmuir 1∶1 model fit. The analyte concentrations corresponding to the curve series are shown to the right of each graph in namolar units. (TIF) [file ppat.1002806.s001.tif]

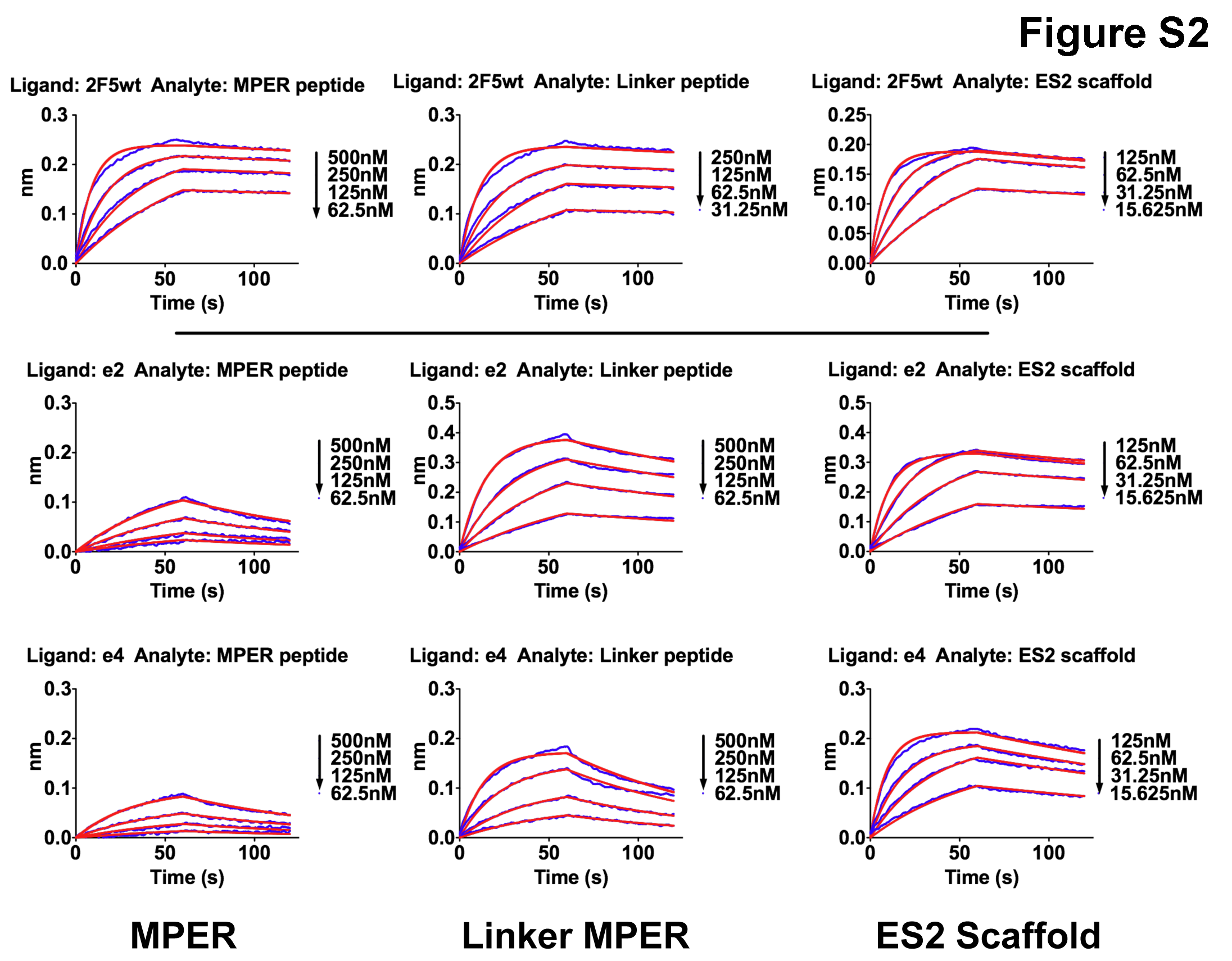

Supplement: Figure S2 — Octet binding curves of elongated CDRH3 antibodies to selected MPER analytes. Displayed on top of each graph are the name of the ligand Mab and the corresponding MPER analyte. In blue are the experimental curves and in red the curves corresponding to the applied Langmuir 1∶1 model fit. The analyte concentrations corresponding to the curve series are shown to the right of each graph in namolar units. (TIF) [file ppat.1002806.s002.tif]

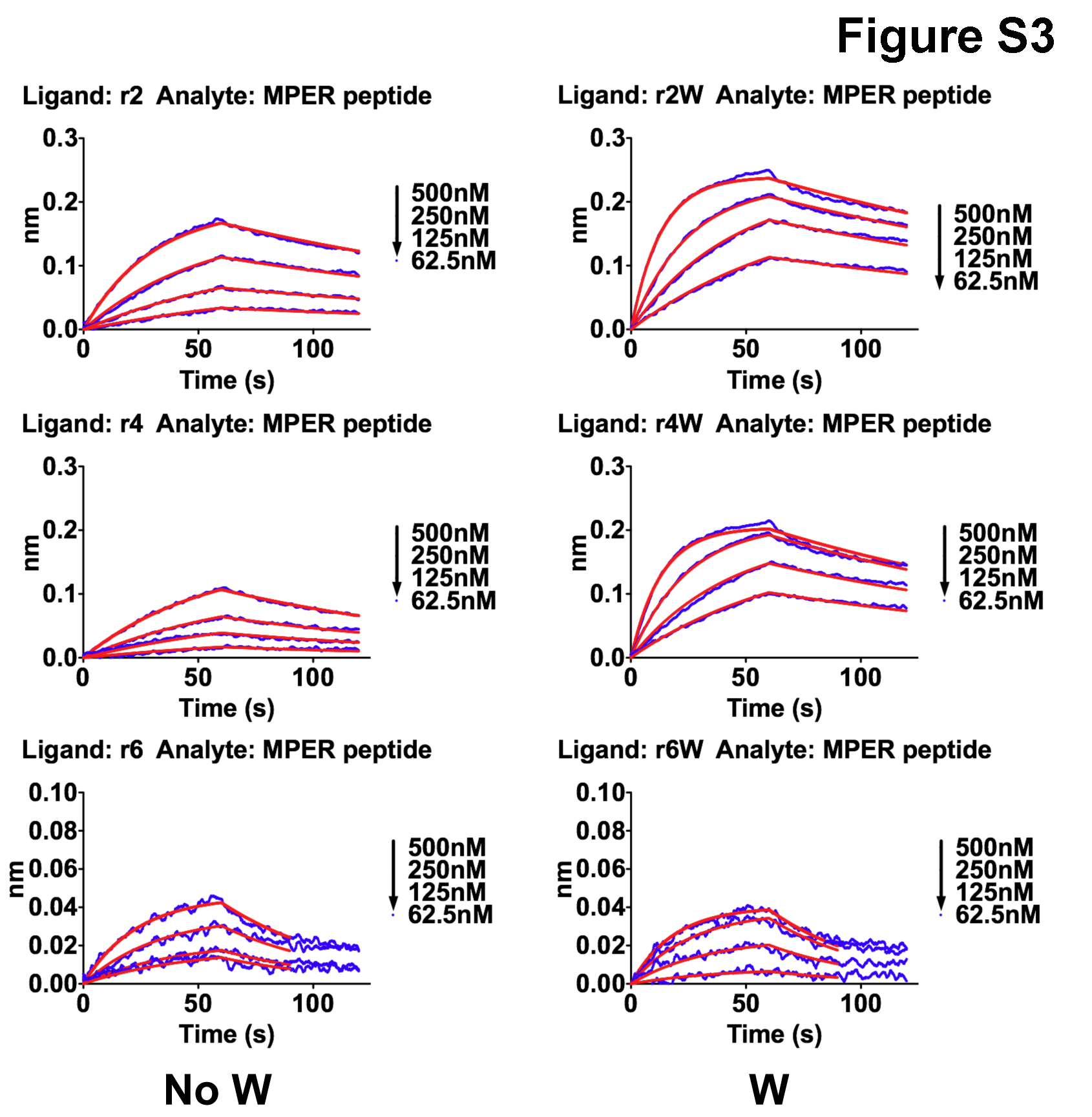

Supplement: Figure S3 — Effect of W substitutions on binding to the MPER peptide by reduced CDRH3 length antibodies. The graphs are organized in pairs to show the effects of the W substitution on the antibody binding to the MPER peptide before (left) and after the W substitution (right). In blue are the experimental curves and in red the curves corresponding to the applied Langmuir 1∶1 model fit. The analyte concentrations corresponding to the curve series are shown to the right of each graph in namolar units. (TIF) [file ppat.1002806.s003.tif]

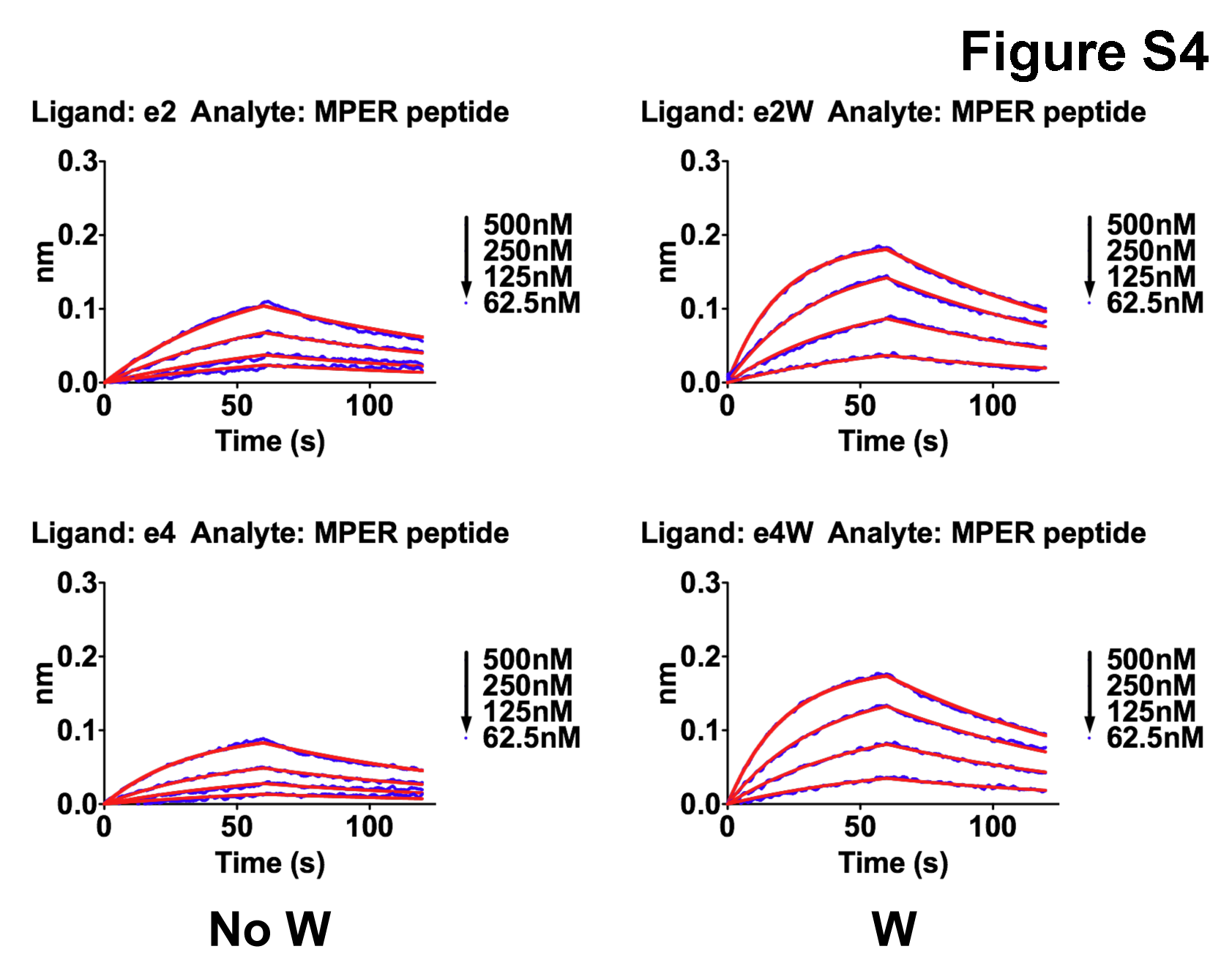

Supplement: Figure S4 — Effect of W substitutions on binding to the MPER peptide by elongated CDRH3 antibodies. The graphs are organized in pairs to show the effects of the W substitution on the antibody binding to the MPER peptide before (left) and after the W substitution (right). In blue are the experimental curves and in red the curves corresponding to the applied Langmuir 1∶1 model fit. The analyte concentrations corresponding to the curve series are shown to the right of each graph in namolar units. (TIF) [file ppat.1002806.s004.tif]

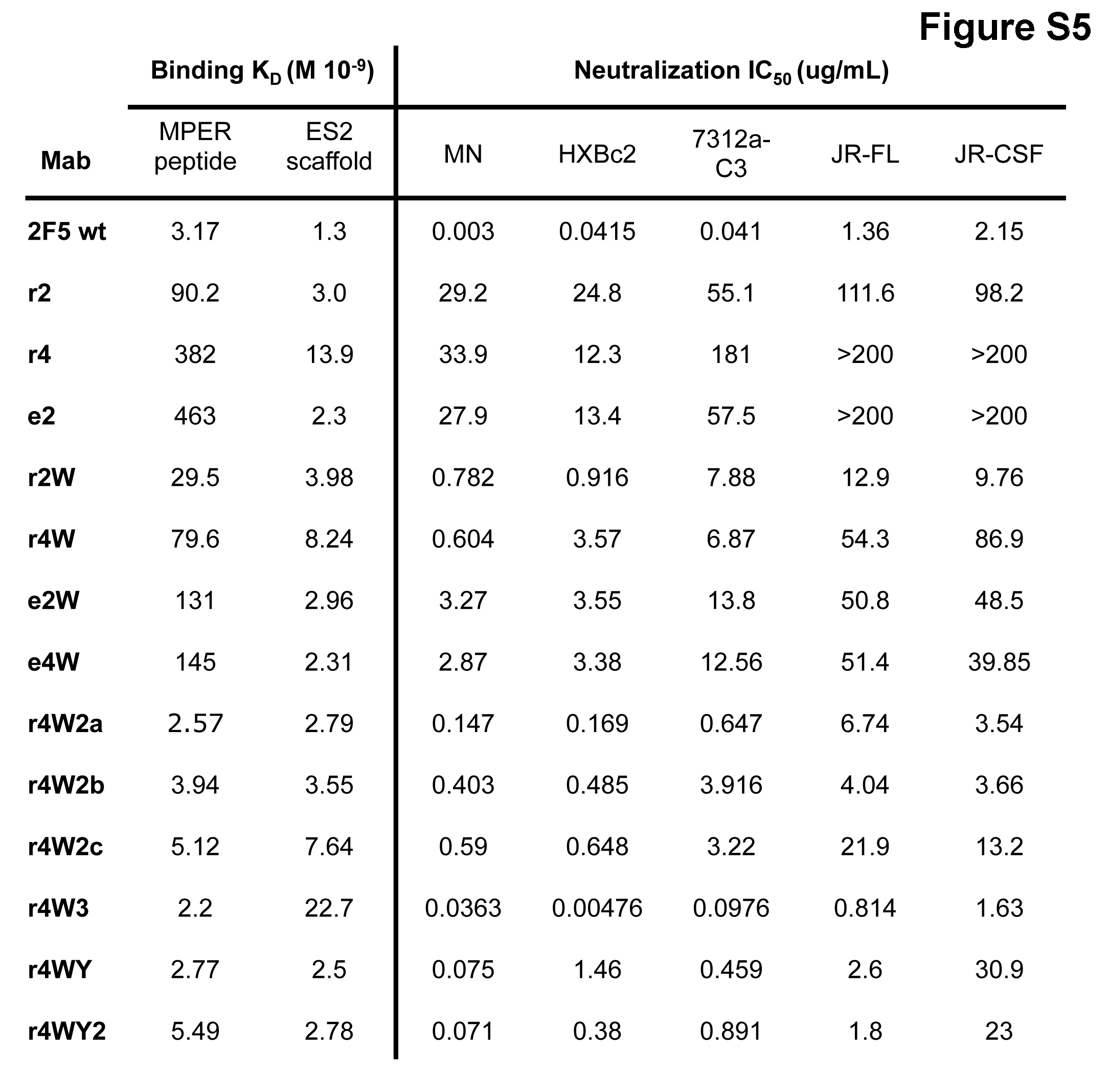

Supplement: Figure S5 — Binding affinity constants to wt MPER peptide and ES2 scaffold and Mab neutralization IC50s. The table displays the values used to calculate the correlation between binding and neutralization in Figure 5. Mab affinities (KD) to the wt MPER peptide and the ES2 scaffolds are shown along side with the IC50s for a panel of five pseudoviruses. (TIF) [file ppat.1002806.s005.tif]

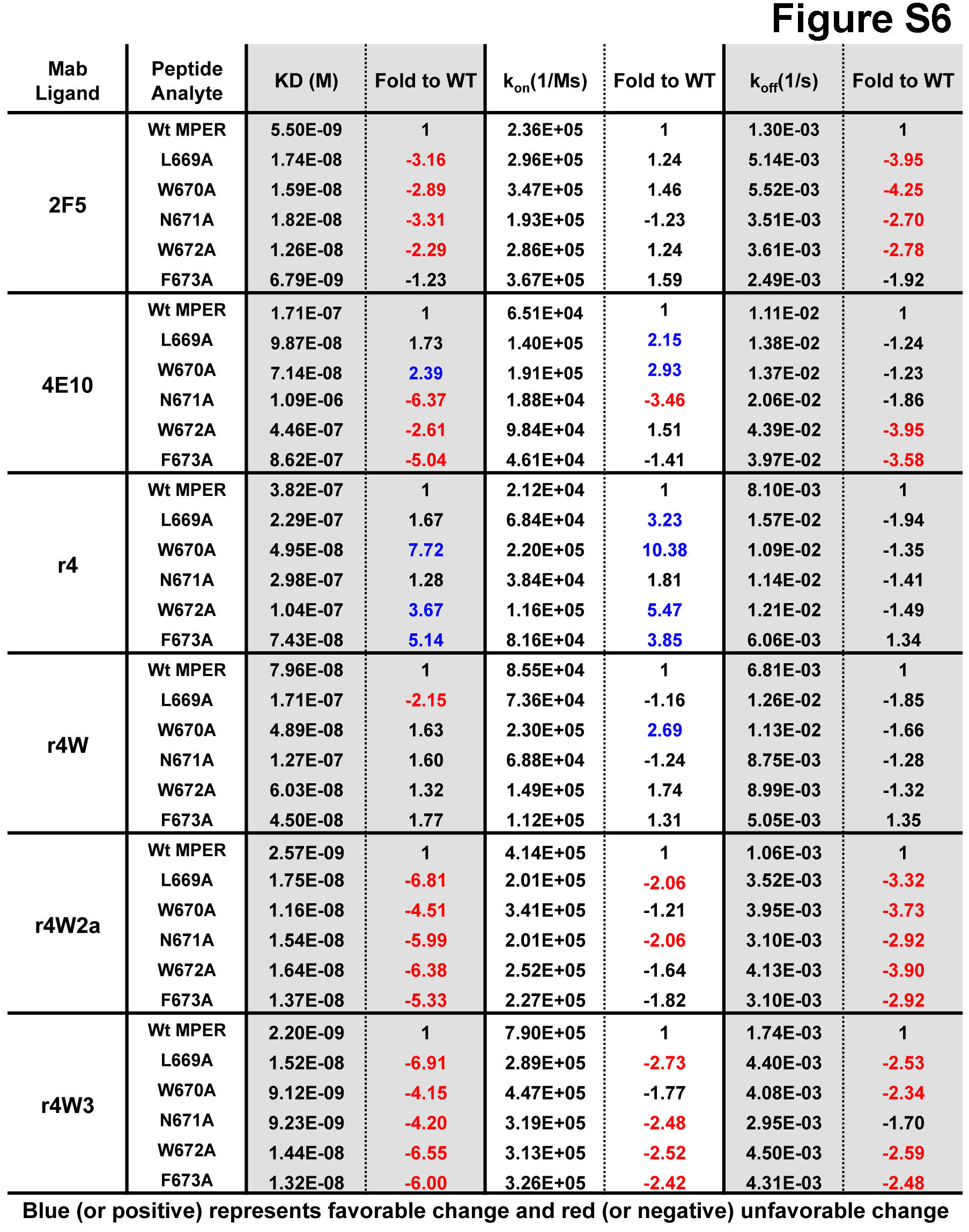

Supplement: Figure S6 — Binding kinetic constants to alanine substituted MPER peptides. Shown in this table are the affinity constant (KD), the on-rate (kon) and off-rate (koff) for the Mabs used in the alanine scanning analysis. Next to each parameter is the corresponding fold-change with respect to antibody binding to wt MPER peptide. A red/negative number represents an unfavorable change (i.e.: decrease in affinity or decrease in on-rate or a faster off-rate) whereas a blue/positive number represents a favorable change. (TIF) [file ppat.1002806.s006.tif]

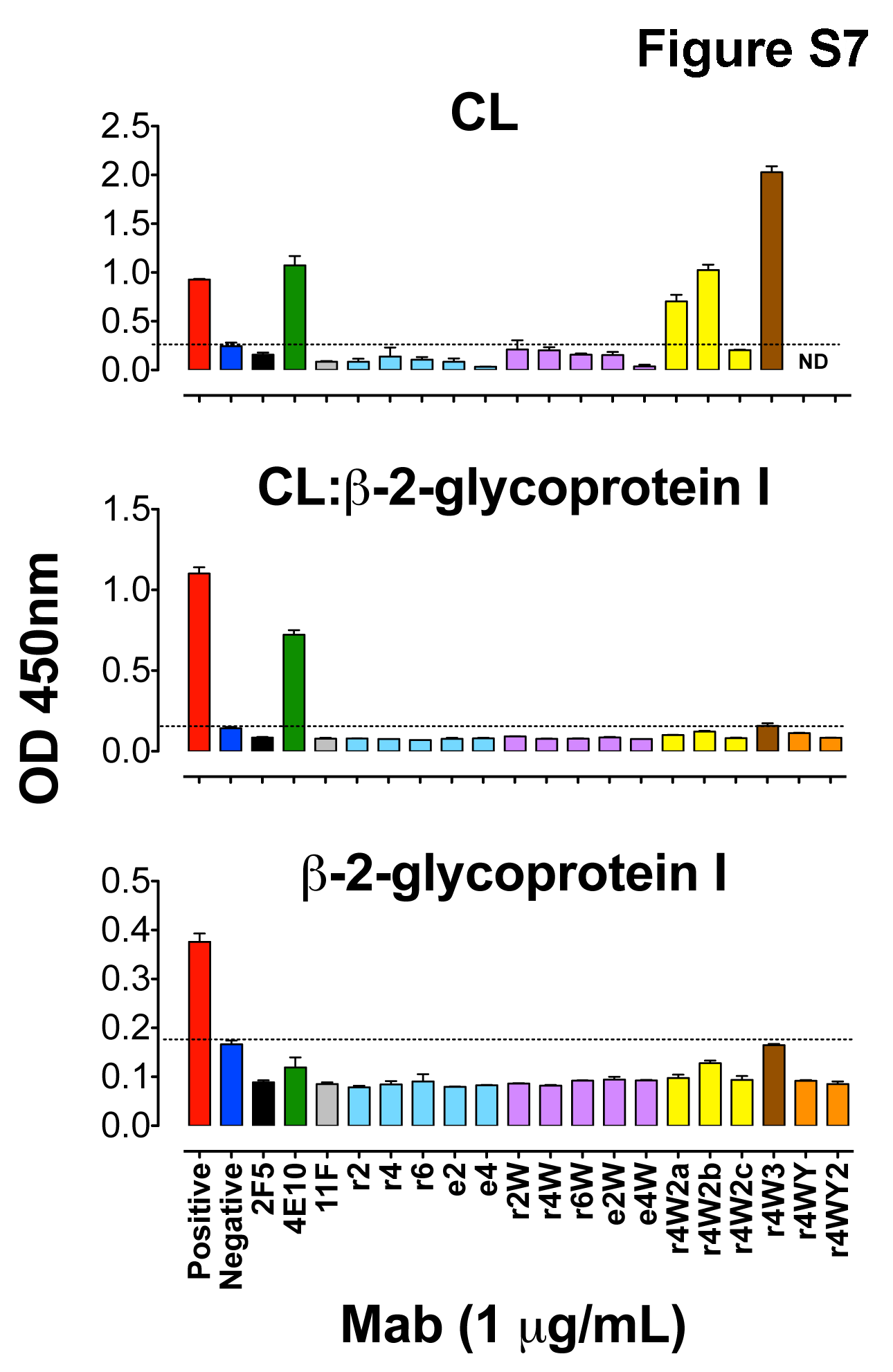

Supplement: Figure S7 — Antibody binding to CL:beta-2-glycoprotein I. Bars represent ELISA optical density readings at 450 nm wavelength corresponding to binding of antibodies (2F5 in black, 4E10 in green, 11F in gray and the CDRH3 altered variants in blue, violet, yellow, brown and purple) at 1 µg/mL concentration to CL (top panel), CL:beta-2glycoprotein I complex (middle) and beta-2glycoprotein I (bottom). Blue color designates CDRH3 altered antibodies with no W substitutions, violet with one W substitution, yellow with two W substitutions, brown with three W substitutions, and purple with one W and one or two Y substitutions. (TIF) [file ppat.1002806.s007.tif]
